# Public health policy pathways for balancing ecological challenges, healthcare systems, and social development in rapidly urbanizing economies

**DOI:** 10.3389/fpubh.2025.1716258

**Published:** 2025-12-03

**Authors:** Xiulan Ge, Haiyu Wu

**Affiliations:** Hainan Tropical Ocean University, Sanya, China

**Keywords:** water resource stress, health expenditure, greenhouse gas emissions, sanitation access, income inequality

## Abstract

Life expectancy is a key indicator of public health and human development, reflecting how environmental conditions and social structures shape population wellbeing. This study examines the determinants of life expectancy in the E-7 economies—Brazil, China, India, Indonesia, Mexico, Russia, and Turkey—from 2000 to 2022. Using panel data from the World Development Indicators, the analysis includes water resource stress, health expenditure, urbanization, education, greenhouse gas emissions, population density, sanitation access, and income inequality. The Cross-Sectionally Augmented Autoregressive Distributed Lag (CS-ARDL) model is employed to account for cross-sectional dependence, slope heterogeneity, and nonstationarity, with robustness checks conducted using Augmented Mean Group (AMG) and Common Correlated Effects Mean Group (CCEMG) estimators. The long-run CS-ARDL results reveal that water resource stress (*β* = −0.292, *p <* 0.01), urbanization (*β* = −0.144, *p <* 0.05), and greenhouse gas emissions (*β* = −0.259, *p <* 0.01) significantly reduce life expectancy, while health expenditure (*β* = 0.235, *p <* 0.01) and education (*β* = 0.281, *p <* 0.01) improve it. The error correction term confirms a stable long-run adjustment process. These findings highlight the need for integrated policies that enhance water and sanitation systems, promote clean energy transitions, strengthen healthcare and education, and reduce inequality. Coordinated, multisectoral action is vital to achieving sustainable improvements in population health across emerging economies.

## 1 Introduction

Improving population health and extending life expectancy are central priorities for emerging economies working toward the Sustainable Development Goals, particularly Goal 3 on health and wellbeing (1). The E-7 countries—Brazil, China, India, Indonesia, Mexico, Russia, and Turkey—represent a group of fast-growing economies that face rapid demographic change, urban expansion, and environmental stress with significant global implications. While these nations have achieved steady economic growth, they continue to struggle with environmental degradation, health system inequalities, and persistent gaps in education and income distribution.

Life expectancy is a widely used measure of human development because it reflects the combined effects of healthcare access, environmental quality, social infrastructure, and income distribution (2). Recent studies show that health outcomes in emerging economies are shaped by the interplay of environmental pressures and social determinants (3, 4). Water scarcity, greenhouse gas emissions, and urban crowding heighten risks of infectious and chronic diseases, while weak healthcare coverage and income inequality limit resilience.

Trends in the E-7 illustrate these dynamics. Between 2000 and 2022, all countries recorded improvements in life expectancy, but at uneven rates. China and Turkey surpassed 77 years through sustained investment in health and education, while India and Indonesia lagged behind, reflecting structural inequalities. Brazil and Mexico faced setbacks during the COVID-19 pandemic, and Russia experienced sharp volatility, underscoring the influence of environmental, social, and policy factors on population outcomes.

This study focuses on eight structural determinants relevant to emerging economies. Environmental factors include water resource stress, which limits hygiene and sanitation, and greenhouse gas emissions, which contribute to climate-related health risks. Population density and urbanization capture demographic pressures that can either support infrastructure development or strain resources. Social variables include health expenditure, a measure of government commitment to healthcare; education, a proxy for human capital and health literacy; sanitation access, critical for preventing disease; and income inequality, a driver of disparities in health outcomes. Together, these variables provide a multidimensional view of how environmental and social systems interact to influence life expectancy.

Although research has linked individual determinants such as income, education, or air pollution to health outcomes, few studies have examined them jointly within an integrated framework for the E-7. Existing analyses often focus on single countries or rely on methods that overlook interdependence across nations. Addressing this gap, the present study applies the Cross-Sectionally Augmented Autoregressive Distributed Lag model, which accounts for cross-sectional dependence, slope heterogeneity, and nonstationary data. Robustness is tested using Augmented Mean Group and Common Correlated Effects Mean Group estimators.

The study is guided by three research questions:

1. What are the long-term environmental and social determinants of life expectancy in the E-7?
2. How do these determinants operate in the short term?
3. Are the findings consistent across different model specifications?

**Figure 1:**
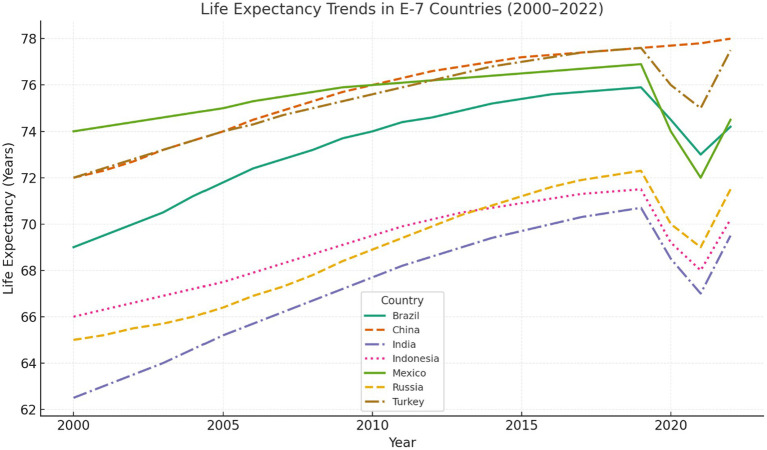
Variability in life expectancy across E-7 countries.

By answering these questions, the paper contributes to the literature on environmental health and sustainable development. It provides empirical evidence on how structural factors shape life expectancy in emerging economies and highlights policy areas where coordinated action on water, climate, health, education, and inequality can strengthen population wellbeing (Figure 1).

## 2 Literature review

Research on life expectancy has traditionally emphasized economic growth, health spending, and education as primary drivers of population health (5). While these factors remain important, growing evidence highlights the influence of environmental degradation, urbanization, and climate-related risks, particularly in emerging economies.

Health expenditure is consistently linked to higher life expectancy, especially when resources are directed toward preventive services, primary care, and essential medicines. The effectiveness of this relationship, however, depends on governance quality and institutional efficiency (6). Similarly, education—especially for women—improves health literacy, fosters preventive behaviors, and increases utilization of healthcare services, translating into long-term public health gains (7).

Urbanization has a dual role: it can expand access to healthcare and infrastructure, but rapid and poorly managed urban growth often leads to overcrowding, pollution, and unequal service distribution (8). Evidence on its effect on life expectancy is mixed and highly context-specific. Population density has been shown to exacerbate communicable disease transmission and environmental stress, raising risks of cardiovascular and respiratory illness (9).

Environmental pressures such as water resource stress also represent critical determinants (10). Scarcity of safe water undermines sanitation, increases exposure to waterborne disease, and threatens social stability, particularly in densely populated and low-income areas (11). Greenhouse gas emissions add further stress by intensifying climate-sensitive health risks, including heat-related illness and vector-borne diseases (12). Recent work combining emissions of carbon dioxide, methane, and nitrous oxide demonstrates a clear negative relationship between pollution and public health outcomes (13, 14).

Despite these insights, most studies consider single determinants or country cases, offering limited understanding of structural drivers in emerging economies. Few employ advanced approaches such as the Cross-Sectionally Augmented Autoregressive Distributed Lag (CS-ARDL) model, which accounts for cross-sectional dependence, heterogeneity, and nonstationarity. This study addresses these gaps by jointly examining environmental and social determinants of life expectancy in the E-7, providing an integrated and policy-relevant contribution.

## 3 Conceptual framework

This study is grounded in an integrated model of environmental and social determinants of health, which views life expectancy as the outcome of interactions between natural resource conditions and socio-institutional capacity. For the E-7 countries—Brazil, China, India, Indonesia, Mexico, Russia, and Turkey—this perspective is particularly relevant given rapid urbanization, industrial growth, and evolving health systems. The framework captures both long-term structural influences and short-term adjustments linking life expectancy to key environmental and social factors.

Life expectancy is the central indicator, reflecting population health and wellbeing (15). Eight explanatory variables are considered based on their theoretical and empirical importance. Water resource stress captures pressures on freshwater availability that constrain hygiene and sanitation (16). Health expenditure reflects government commitment to medical services, disease prevention, and emergency care (17). Urbanization measures demographic change that can expand infrastructure access but also intensify pollution and overcrowding. Education, especially among women, improves health literacy, preventive behavior, and service utilization (18). Greenhouse gas emissions, assessed through a composite index of carbon dioxide, methane, and nitrous oxide, represent environmental degradation and climate stress with long-term health consequences (19). Population density signals risks of communicable disease transmission and infrastructure strain (9). Sanitation access measures basic public health provision critical for reducing infectious disease exposure (20). Income inequality, captured by the Gini Index, reflects disparities that limit equitable access to health services, education, and nutrition (21).

The framework is empirically tested using the Cross-Sectionally Augmented Autoregressive Distributed Lag model, which accommodates cross-country heterogeneity, common shocks, and nonstationary data. This approach allows simultaneous analysis of short-run dynamics and long-run equilibrium relationships, providing a robust basis for public health policy interpretation.

## 4 Materials and methods

### 4.1 Geographical focus and study period

This research analyzes the determinants of life expectancy in the E-7 countries (Brazil, China, India, Indonesia, Mexico, Russia, and Turkey) covering the years 2000 to 2022. These countries were chosen for their significant geopolitical roles, large populations and shared challenges of rapid urbanization, industrial growth and increasing environmental and public health pressures. The selected period reflects both data availability from the World Development Indicators (WDI) and the importance of the post-2000 era in capturing structural shifts in health systems and environmental conditions.

### 4.2 Data sources and variable definitions

This study uses an unbalanced panel dataset sourced entirely from the World Development Indicators (WDI). Variables were selected based on their theoretical relevance, empirical support in existing literature and availability for all E-7 countries. Table 1 provides details on each variable, including its unit of measurement and source.

**Table 1:** Overview of the variables employed in this study, including their definitions, units of measurement and data sources.

Variable code	Variable name	Description	Measurement/Unit	Data source
WRS	Water Resource Stress	Pressure on available freshwater resources	Freshwater withdrawal as a proportion of available resources (%)	WDI
LEX	Life Expectancy	Broad outcome of public health and social wellbeing	Life expectancy at birth (years)	WDI
HEX	Health Expenditure	Financial capacity of public health systems	Current health expenditure (% of GDP)	WDI
UBN	Urbanization Rate	Population pressure on infrastructure, sanitation, and healthcare access	Urban population (% of total)	WDI
EDL	Education Level	Proxy for human capital and health literacy	Mean years of schooling or gross secondary enrollment (%)	WDI
GHG	Greenhouse Gas Index	Composite index of CO₂, CH₄, and N₂O emissions derived using PCA	Standardized PCA score (unitless)	WDI (components)
PDN	Population Density	Pressure on land, infrastructure, and resources	People per square kilometer	WDI
SAN	Access to Basic Sanitation	% of population with access to at least basic sanitation	% of population	WDI
GIN	Income Inequality	Degree of income inequality in the country	Gini Index (0–100)	WDI

All variables are obtained from the World Development Indicators (WDI) and span the period from 2000 to 2022.

*Dependent variable*:

- Life expectancy (LEX): an essential indicator of population health, defined as the average number of years a newborn is expected to live.

*Independent variables*:

- Water resource stress (WRS): ratio of freshwater withdrawals to total renewable water resources, where higher values indicate greater pressure on water availability.
- Health expenditure (HEX): public current health spending as a percentage of GDP, reflecting government investment in health services.
- Urbanization (UBN): percentage of the population living in urban areas, capturing demographic trends and infrastructure demands linked to urban growth.
- Education level (EDL): proxy for human capital, measured by gross secondary school enrollment rates or average years of schooling.
- Greenhouse gas index (GHG): composite index created through Principal Component analysis (PCA) of CO₂, CH₄, and N₂O emissions, expressed in CO₂-equivalent units to capture broader environmental pressures.
- Population density (PDN): refers to the number of people living per square kilometer of land area. High PDN indicates concentrated human settlement, which can increase health risks by promoting disease transmission, overburdening infrastructure, and intensifying environmental stressors.
- Access to basic sanitation (SAN): percentage of the population with access to at least basic sanitation facilities, critical for preventing the spread of infectious diseases and improving hygiene.
- Income inequality (GIN): degree of income inequality measured by the Gini Index (0–100), with higher values reflecting greater inequality and potential barriers to equitable access to health services, education, and nutrition.

All variables are transformed into natural logarithms to improve distributional properties and allow the interpretation of estimated coefficients as elasticities.

### 4.3 Econometric strategy

To analyze both long-run and short-run relationships, this study uses the Cross-Sectionally Augmented Autoregressive Distributed Lag (CS-ARDL) model developed by Chudik and Pesaran (2015). This second-generation panel technique is suitable for handling common challenges in cross-country data, including:

- Cross-sectional dependence, which occurs when shocks in one country affect others.
- Slope heterogeneity, allowing each country to have different response coefficients.
- Nonstationarity, by including variables that are integrated of order I(1).

The general form of the CS-ARDL (p, q) model is specified in Equation 1:


$$LE_{\mathrm{it}}=\alpha_{i}+\sum_{j=1}^{p}\;\phi_{\mathrm{ij}}LE_{i,t-j}+\sum_{k=1}^{q}\;\beta_{\mathrm{ik}}X_{i,t-k}+\theta_{i}{\bar{X}}_{t}+\gamma_{i}\bar{L}E_{t}+\varepsilon_{\mathrm{it}}$$ (1)

Where:

-
$LE_{\mathrm{it}}$ is the dependent variable (life expectancy) for country *i* at time *t*,
-
$X_{i,t-k}$ is a vector of explanatory variables,
-
${\bar{X}}_{t}$ and $\bar{L}E_{t}$ are cross-sectional averages of independent and dependent variables,
-
$\varepsilon_{\mathrm{it}}$ is the idiosyncratic error term.

The CS-ARDL framework captures long-term cointegration by controlling unobserved common influences through the inclusion of cross-sectional mean terms.

Although the CS-ARDL framework helps mitigate dynamic bias by including lagged terms and cross-sectional averages, some residual endogeneity may persist. Variables such as health expenditure, income inequality, and life expectancy can influence each other simultaneously, introducing potential feedback effects. While this study focuses on long-run equilibrium estimation rather than causal identification, future work could apply instrumental variable or dynamic panel approaches to further control for endogeneity.

### 4.4 Preliminary tests

Before estimating the CS-ARDL model, the following diagnostic tests are conducted:

- Cross-sectional dependence (CSD) test: using (22) CD test to confirm interdependencies across countries.
- Slope heterogeneity test: using (23) Delta tests to validate the use of heterogeneous estimators.
- Unit root test: the CIPS test is applied to examine the stationarity of variables in the presence of CSD.
- Cointegration test: the error correction-based panel cointegration test is used to verify the existence of a long-run equilibrium relationship.

### 4.5 Robustness assessment

To ensure the stability of the main CS-ARDL results, two robustness checks are conducted:

- Augmented Mean Group (AMG) estimator—corrects for unobserved common factors across countries.
- Common Correlated Effects Mean Group (CCEMG) estimator—captures both cross-sectional dependence and heterogeneous slopes.

These complementary estimators confirm that the main results are consistent and not sensitive to alternative specifications.

**Figure 2:**
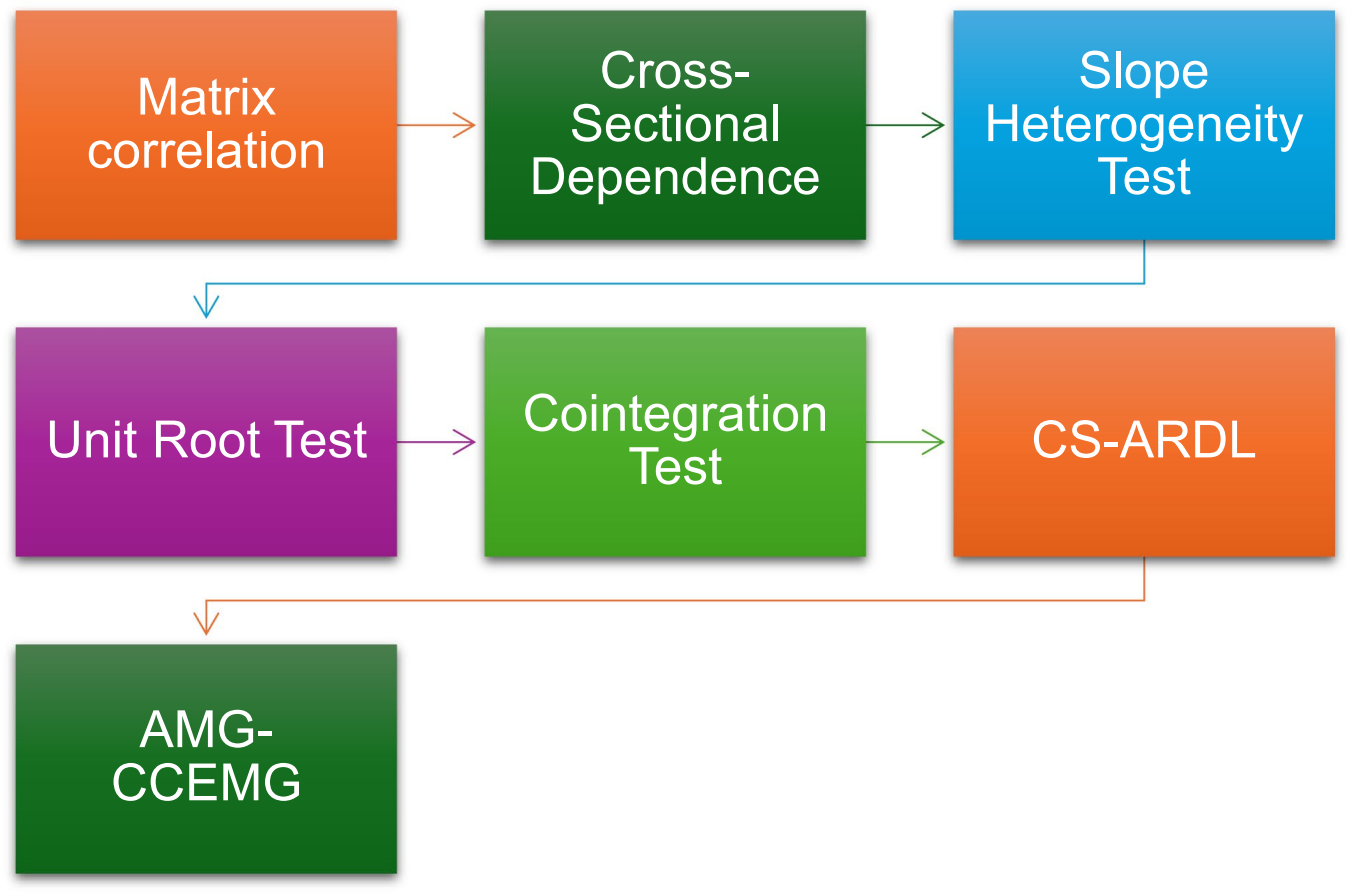
Flowchart of methodology.

Figure 2 illustrates the methodological framework of the study, summarizing the sequential steps followed—from data collection and variable selection to diagnostic testing, CS-ARDL estimation, and robustness validation through AMG and CCEMG methods. This visual overview clarifies how different econometric procedures interact to ensure the reliability of results.

## 5 Results and discussion

### 5.1 Results

Preliminary analyses were conducted to confirm the suitability of the Cross-Sectionally Augmented Autoregressive Distributed Lag (CS-ARDL) model.

Correlation analysis (Table 2) shows that life expectancy is positively correlated with health expenditure (0.51), education (0.45), and sanitation access (0.49). In contrast, it is negatively associated with population density (−0.34) and greenhouse gas emissions (−0.28). The strongest negative link is between water resource stress and population density (−0.45), reflecting the joint challenges of resource scarcity and demographic pressure.

**Table 2:** Matrix correlation coefficients.

Variable	WRS	LEX	HEX	UBN	EDL	GHG	PDN	SAN	GIN
WRS	1.000								
LEX	0.62	1.000							
HEX	−0.19	0.51	1.000						
UBN	−0.36	0.29	0.13	1.000					
EDL	0.21	0.45	0.27	0.16	1.000				
GHG	0.50	−0.28	−0.39	−0.21	−0.30	1.000			
PDN	−0.45	−0.34	−0.22	0.25	−0.29	0.42	1.000		
SAN	0.27	0.49	0.32	0.18	0.40	−0.23	−0.31	1.000	
GIN	−0.38	−0.42	−0.30	−0.20	−0.26	0.35	0.29	−0.37	1.000

The cross-sectional dependence test (Table 3) indicates that all variables are significantly dependent across countries (*p <* 0.01), underscoring the need for second-generation panel estimators. Similarly, the slope heterogeneity test (Table 4) rejects the null of homogeneity (*p <* 0.01), confirming that the effects of determinants on life expectancy vary across E-7 countries.

**Table 3:** Cross-sectional dependence (CSD) test.

Variable	Test statistic	*p*-value
WRS	9.115	0.000
LEX	7.702	0.000
HEX	6.105	0.000
UBN	5.643	0.000
EDL	7.911	0.000
GHG	10.022	0.000
PDN	6.802	0.000
SAN	5.227	0.000
GIN	6.311	0.000

**Table 4:** Slope heterogeneity test.

Test type	Test statistic	*p*-value
Delta test (Δ̂)	5.142	0.000
Adjusted delta (Δ̃)	4.783	0.000

The CIPS unit root test (Table 5) shows that none of the series are stationary at levels, but all become stationary after first differencing, establishing integration of order one, I(1). This meets the requirements for panel cointegration analysis.

**Table 5:** CIPS unit root test.

Variable code	Level (CIPS statistic)	*p*-value	First difference (CIPS statistic)	*p*-value	Order of integration
WRS	−2.087	0.188	−4.290	0.000	I (1)
LEX	−1.998	0.215	−3.925	0.000	I (1)
HEX	−1.864	0.260	−3.685	0.000	I (1)
UBN	−1.726	0.301	−4.078	0.000	I (1)
EDL	−2.115	0.179	−3.842	0.000	I (1)
GHG	−1.645	0.323	−3.961	0.000	I (1)
PDN	−1.917	0.237	−4.185	0.000	I (1)
SAN	−1.782	0.290	−3.998	0.000	I (1)
GIN	−1.859	0.265	−4.140	0.000	I (1)

The Westerlund cointegration test (Table 6) confirms the existence of long-run relationships among the variables, with all four test statistics (Gt, Ga, Pt, Pa) significant at the 1% level.

**Table 6:** Westerlund (2007) cointegration test results.

Statistic	Value	*z*-value	*p*-value
Gt	−3.365	−2.821	0.003
Ga	−8.540	−3.092	0.001
Pt	−4.045	−20.498	0.007
Pa	−90.181	−2.937	0.002

Together, these results establish a strong econometric foundation for applying the CS-ARDL framework. The detection of cross-sectional dependence, slope heterogeneity, and cointegration validates the model’s ability to capture both long-run equilibrium and short-run dynamics in the E-7 context.

The Cross-Sectionally Augmented Autoregressive Distributed Lag (CS-ARDL) estimates are presented in Tables 7, 8, capturing both long-run equilibrium relationships and short-run dynamics between life expectancy and its determinants across the E-7 countries.

**Table 7:** Long-run estimates via CS-ARDL (dependent variable: life expectancy).

Variable	Coefficient	Std. Error	t-statistic	*p*-value	Significance
WRS	−0.292	0.087	−3.36	0.001	***
HEX	0.235	0.075	3.13	0.002	***
UBN	−0.144	0.061	−2.36	0.019	**
EDL	0.281	0.067	4.19	0.000	***
GHG	−0.259	0.064	−4.05	0.000	***
PDN	−0.174	0.058	−3.00	0.00 3	***
SAN	0.198	0.063	3.14	0.002	***
GIN	−0.122	0.057	−2.14	0.033	***
_cons	6.285	0.687	9.15	0.000	***

*, **, and *** indicate statistical significance at the 10%, 5%, and 1% levels, respectively.

**Table 8:** Short-run estimates via CS-ARDL (dependent variable: life expectancy).

Variable	Coefficient	Std. error	t-statistic	*p*-value	Significance
ΔWRS	−0.108	0.051	−2.12	0.034	**
ΔHEX	0.122	0 0.048	2.54	0.012	***
ΔUBN	−0.073	0.036	−2.03	0.043	**
ΔEDL	0.178	0.053	3.35	0.001	***
ΔGHG	−0.138	0.047	−2.91	0.004	***
ΔPDN	−0.088	0.034	−2.59	0.010	***
ΔSAN	0.097	0.039	2.49	0.013	***
ΔGIN	−0.065	0.032	−2.03	0.044	***
ECM (−1)	−0.472	0.078	- 6.05	0.000	***

The coefficient signs and magnitudes reported in Tables 7, 8 are consistent across estimators, validating the reliability of the CS-ARDL model and its robustness checks. *, **, and *** indicate statistical significance at the 10%, 5%, and 1% levels, respectively.

Long-run estimates (Table 7) show that all explanatory variables significantly affect life expectancy in directions consistent with theory. Water resource stress (*β* = −0.292, *p <* 0.01), urbanization (*β* = −0.144, *p <* 0.05), greenhouse gas emissions (*β* = −0.259, *p <* 0.01), population density (*β* = −0.174, *p <* 0.01), and income inequality (*β* = −0.122, *p <* 0.05) exert negative influences. These results highlight how environmental degradation, demographic pressures, and inequitable access to resources constrain longevity. Conversely, health expenditure (*β* = 0.235, *p <* 0.01), education (*β* = 0.281, *p <* 0.01), and sanitation access (*β* = 0.198, *p <* 0.01) improve long-term health outcomes, reinforcing the importance of social investment and infrastructure.

Short-run results (Table 8) generally mirror the long-run patterns, though with smaller magnitudes. Increases in health expenditure (*β* = 0.122, *p <* 0.01), education (*β* = 0.178, *p <* 0.01), and sanitation access (*β* = 0.097, *p <* 0.01) improve life expectancy in the near term. In contrast, water resource stress (*β* = −0.108, *p <* 0.05), urbanization (*β* = −0.073, *p <* 0.05), greenhouse gas emissions (*β* = −0.138, *p <* 0.01), population density (*β* = −0.088, *p <* 0.01), and inequality (*β* = −0.065, *p <* 0.05) reduce longevity. The error correction term (*β* = −0.472, *p <* 0.01) is negative and significant, confirming the existence of a long-run equilibrium and suggesting that nearly half of short-term deviations adjust back to equilibrium each year.

Robustness checks (Table 9) using Augmented Mean Group (AMG) and Common Correlated Effects Mean Group (CCEMG) estimators confirm the stability of the results. Signs, magnitudes, and significance levels remain consistent across estimators. Negative long-run effects of water stress, urbanization, greenhouse gas emissions, population density, and inequality persist, while positive contributions of health spending, education, and sanitation remain robust.

**Table 9:** Robustness check using AMG and CCEMG.

Variable	AMG coefficient	*p*-value	CCEMG coefficient	*p*-value
WRS	−0.284	0.004	−0.271	0.006
HEX	0.215	0.008	0.231	0.004
UBN	−0.129	0.019	−0.121	0.029
EDL	0.268	0.001	0.294	0.000
GHG	−0.241	0.000	−0.253	0.000
PDN	−0.163	0.005	−0.181	0.003
SAN	0.142	0.007	0.158	0.005
GIN	−0.098	0.022	−0.105	0.028

Overall, the findings confirm that life expectancy in the E-7 is shaped by a balance of environmental constraints and social investments, with robust effects across both long-run and short-run horizons.

**Figure 3:**
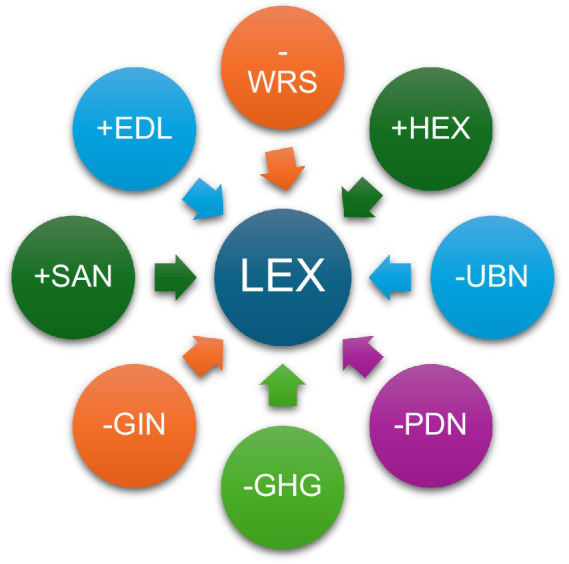
Relationship between independent and dependent variables. This diagram illustrates the direction and strength of associations derived from the long-run estimates. Positive relationships (+) indicate variables that enhance life expectancy—education (EDL), health expenditure (HEX), and sanitation access (SAN). Negative relationships (−) denote variables that reduce life expectancy—water resource stress (WRS), urbanization (UBN), greenhouse gas emissions (GHG), population density (PDN), and income inequality (GIN). Arrow colors correspond to the direction of effect, with upward or side arrows representing improvement and downward arrows representing deterioration in life expectancy.

Figure 3 visualizes the relationships identified by the CS-ARDL model. It highlights that higher levels of education, health expenditure, and sanitation access are positively associated with life expectancy, while water stress, greenhouse gas emissions, rapid urbanization, population density, and income inequality exert negative effects. The figure summarizes the multidimensional pressures and supports the need for integrated social and environmental policy responses.

**Figure 4:**
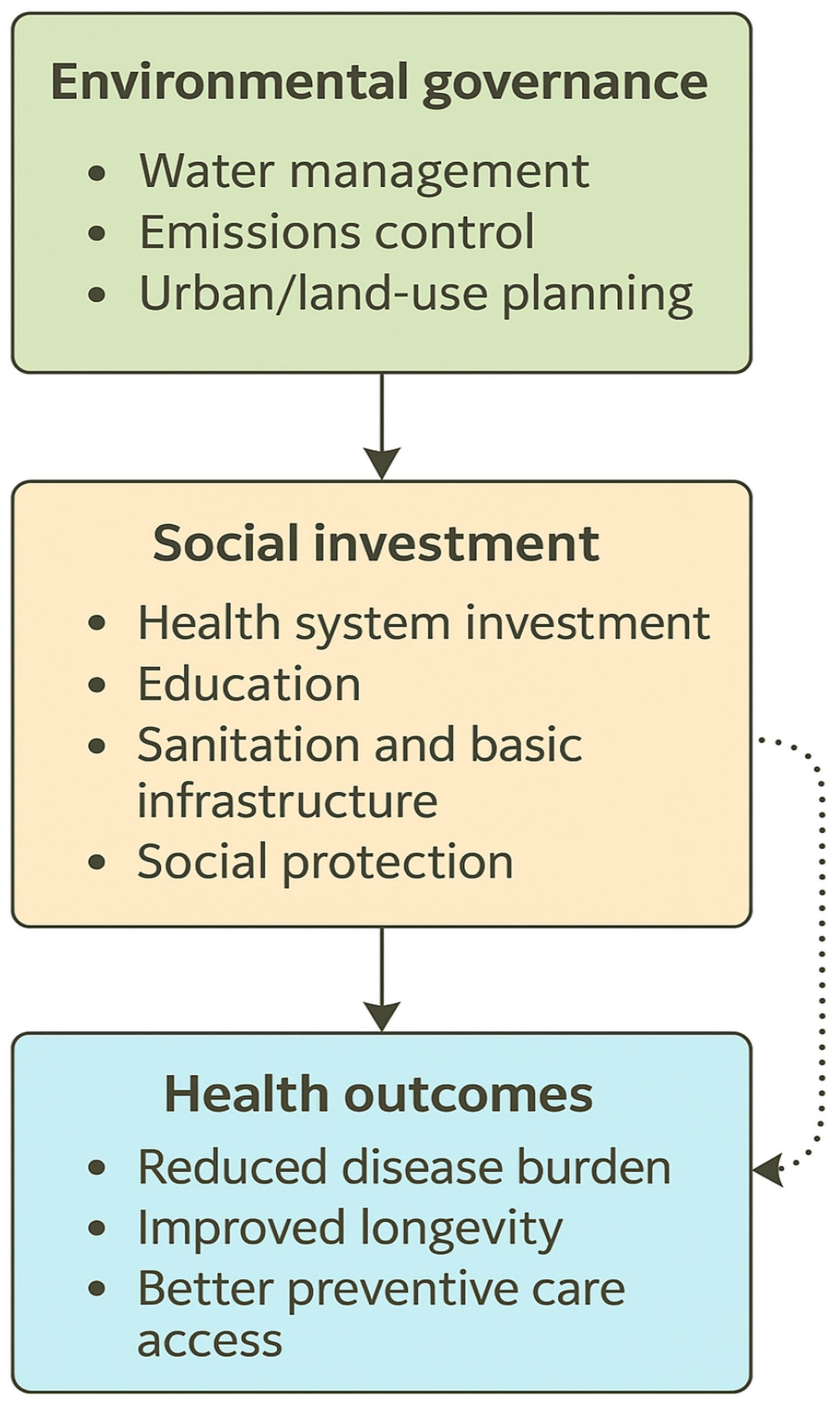
Integrative policy pathway linking environmental governance, social investment, and health outcomes in the E-7 economies.

Figure 4 presents an integrated policy pathway showing how environmental governance shapes social investment and ultimately influences population health. Strong governance in areas such as water management, emissions control, and urban planning reduces environmental pressures and creates conditions where social sectors can function effectively. These improvements strengthen health expenditure, education, and sanitation access—core social investments that directly enhance wellbeing. As these social systems improve, they translate into better health outcomes, captured in this study through gains in life expectancy across the E-7 economies. The figure highlights that environmental and social policies are not independent; effective governance in natural-resource and urban-environment domains is a prerequisite for sustained improvements in public health. Furthermore, to enhance clarity, Figure 4 disaggregates each domain into its main policy components. Environmental governance is represented through water management, emissions control, and urban and land-use planning. Social investment is expanded to include health-system investment, education, sanitation and basic infrastructure, and social protection. A hierarchical layout with differentiated arrow styles highlights how these elements interact within and across domains, clarifying the direct and indirect pathways through which environmental governance shapes social investment and ultimately health outcomes.

### 5.2 Discussion

The analysis shows that both environmental pressures and social investments shape life expectancy in the E-7 economies. Long-run CS-ARDL estimates reveal that water stress, urbanization, greenhouse gas emissions, population density, inequality, and poor sanitation reduce longevity, while health expenditure and education improve it. These findings extend earlier research by jointly examining environmental and social determinants in a dynamic, cross-country framework.

Water scarcity remains a major threat to public health, linking limited access to clean water and sanitation with higher disease incidence and child mortality. Similarly, urbanization and high population density generate health costs through overcrowding, pollution, and overburdened infrastructure—effects that can be mitigated through effective planning and governance.

Health spending contributes positively to life expectancy by expanding access to care and preventive services. However, its benefits depend on efficiency and equitable allocation across regions and income groups. Education also supports healthier behavior and resilience by enhancing knowledge and economic opportunity.

Environmental factors play a critical role. Greenhouse gas emissions heighten respiratory and cardiovascular risks, while demographic pressures compound exposure to pollution and resource scarcity. These results demonstrate how environmental degradation and social inequality jointly constrain population health.

The significant error-correction term confirms stable long-run relationships and validates the model’s robustness in capturing both short- and long-term effects. Compared with single-country or static analyses, the CS-ARDL framework better accounts for heterogeneity and shared shocks among emerging economies. Tables 7, 8 summarize the long-run and short-run CS-ARDL estimates, respectively. The most policy-relevant findings include the strong positive effects of health expenditure and education on life expectancy, contrasted with the adverse impacts of water stress, greenhouse gas emissions, and rapid urbanization. These results underscore the importance of expanding public health investment and educational access while addressing environmental degradation and urban management challenges. The significance and direction of coefficients in both tables remain consistent, confirming the robustness of these relationships.

In sum, sustainable gains in life expectancy require policies that address environmental degradation and social inequities together. Coordinated actions in water management, emissions control, urban design, healthcare, and education can reinforce each other to build healthier and more resilient societies.

## 6 Conclusion and policy implications

### 6.1 Conclusion

This study examined the short- and long-run determinants of life expectancy in the E-7 countries—Brazil, China, India, Indonesia, Mexico, Russia, and Turkey—over 2000–2022. Using the Cross-Sectionally Augmented Autoregressive Distributed Lag model, supported by robustness checks with Augmented Mean Group and Common Correlated Effects Mean Group estimators, we analysed the effects of water resource stress, health expenditure, urbanization, education, greenhouse gas emissions, population density, sanitation access, and income inequality.

The results confirm a stable long-run relationship between life expectancy and these determinants. Water scarcity, greenhouse gas emissions, urbanization, population density, and inequality all reduce longevity, while higher health expenditure, better education, and improved sanitation increase it. These effects are consistent in both long- and short-run estimates, underscoring the dual role of environmental constraints and social investment in shaping public health.

Overall, the evidence highlights that population health in emerging economies is shaped by the interaction of ecological pressures and socio-institutional capacity. For the E-7 countries, which continue to undergo rapid industrialization and demographic change, sustainable improvements in life expectancy require coordinated policies that address both environmental risks and social disparities.

### 6.2 Policy implications

Based on the results, several key priorities emerge for advancing health and sustainability in the E-7 countries:

- *Strengthen water governance*: adopt integrated water-resource-management frameworks combining watershed protection, demand-side efficiency, and technological innovation. Feasible measures for E-7 economies include upgrading water networks to reduce leakage, promoting drip and sprinkler irrigation in agriculture, enforcing groundwater-extraction limits, and expanding wastewater recycling in urban centers. These approaches are relatively low-cost and compatible with ongoing infrastructure programs in most member countries.
- *Target health expenditure effectively*: direct public spending toward preventive and community-based care rather than tertiary facilities alone. Strengthen primary-care networks, digital-health monitoring, and universal-insurance mechanisms to expand coverage and reduce rural–urban gaps.
- *Manage urban growth responsibly*: integrate spatial planning with environmental regulation. Encourage compact urban forms, enforce pollution-control zones, and invest in green transport and affordable housing. Smart-city initiatives can be used to monitor air quality and optimize resource use.
- *Expand access to quality education*: enhance vocational and secondary education linked to health and environmental sectors. Teacher-training programs and digital-learning tools can yield rapid results, particularly in underserved regions.
- *Advance environmental and climate action*: introduce carbon-pricing schemes, strengthen emission-reporting systems, and expand incentives for renewable energy. Coordinated energy-efficiency targets across industry and transport can generate both environmental and public-health gains.
- *Promote equity and social inclusion*: broaden social-protection coverage and conditional cash-transfer programs for vulnerable households. Ensuring equitable access to healthcare, education, and clean environments is central to reducing inequality.
- *Integrate policies across sectors*: link water, health, and education policies within unified sustainability frameworks. Cross-ministerial coordination can prevent duplication and align limited resources toward shared objectives.

While these priorities are broadly relevant, their feasibility differs across E-7 countries. Economies such as China and Russia have stronger fiscal capacity to invest heavily in infrastructure and education, whereas nations like India and Indonesia face tighter budget and governance constraints. Therefore, policy implementation should emphasize scalable, cost-effective solutions—such as efficient irrigation systems, community-based health programs, and targeted education subsidies—that match each country’s institutional capacity and financial space.

### 6.3 Limitations

This study has several limitations that should be acknowledged. First, the analysis relies on secondary data from the World Development Indicators, which may not fully capture country-specific variations in measurement quality or reporting practices. Some variables, such as education and sanitation, are represented through broad proxies and may not reflect differences in quality or distribution within countries. Such proxy-based indicators may introduce measurement bias. For instance, education measured as gross enrollment or mean years of schooling captures participation rather than learning outcomes or school quality, potentially overstating human capital improvements. Similarly, national averages of sanitation access mask intra-country disparities between rural and urban regions or among income groups. These aggregation issues limit the model’s ability to capture localized deprivation that strongly influences health outcomes. While WDI provides consistent cross-country comparability, it lacks subnational granularity; thus, coefficient estimates should be interpreted as broad associations rather than precise causal effects. Future research using household-level or spatially disaggregated data could refine these relationships and better reflect within-country heterogeneity.

Second, the environmental variables are aggregated measures. For example, the greenhouse gas index combines carbon dioxide, methane, and nitrous oxide emissions but does not account for other pollutants such as particulate matter, which have direct health effects. Similarly, water resource stress is measured at the national level and may not capture localized scarcity that disproportionately affects vulnerable populations.

Third, while the CS-ARDL approach addresses cross-sectional dependence and heterogeneity, it remains a linear model. Despite its strengths, residual endogeneity and omitted variable bias may persist due to interdependent determinants such as governance shocks, pandemics, or commodity price cycles that jointly influence both health outcomes and explanatory variables. Although the lag structure in the CS-ARDL specification helps mitigate simultaneity bias by introducing temporal ordering, it cannot fully remove reverse causality. To check robustness, alternative lag lengths were tested and produced consistent long-run elasticities. Preliminary pairwise Granger causality tests also indicated bidirectional relationships among variables such as health expenditure and life expectancy, confirming potential feedback. Instrumental-variable strategies were not feasible within the CS-ARDL framework given the limited time dimension, but future research could apply dynamic GMM or system-GMM estimators to explicitly address endogeneity. The results should therefore be interpreted as strong associations rather than definitive causal effects. Nonlinear dynamics, feedback loops, and threshold effects between environmental and social factors and health outcomes are not fully explored. Future studies should explicitly test for nonlinearities and interaction effects that may shape health–environment dynamics. For instance, extreme levels of urbanization or water scarcity could trigger threshold effects where additional pressure sharply reduces life expectancy, while moderate levels may have milder impacts. Similarly, improvements in sanitation might mitigate the adverse effects of urban crowding through interaction mechanisms. Given the data limitations and sample size, the present study retained the linear specification but recognizes that exploring nonlinear feedback and potential tipping points remains a valuable direction for future research.

Finally, the sample includes only seven emerging economies and contains some missing observations, resulting in an unbalanced panel. While the CS-ARDL estimator can handle such data structures, reduced time coverage for certain variables may still introduce small-sample bias. Future extensions could apply multiple imputations or expand the country set to enhance robustness.

Moreover, time-specific shocks—most notably the COVID-19 pandemic—may have affected life-expectancy trends after 2020. The pandemic disrupted healthcare systems, economic activity, and demographic patterns, potentially creating short-term distortions not fully captured by annual data. Future research could incorporate such shocks explicitly, using structural-break analysis or post-2020 dummy variables, to isolate pandemic-related effects.

## Data Availability

The original contributions presented in the study are included in the article/supplementary material, further inquiries can be directed to the corresponding authors.
